# Electrical Ventricular Remodeling in Dilated Cardiomyopathy

**DOI:** 10.3390/cells10102767

**Published:** 2021-10-15

**Authors:** Christine Mages, Heike Gampp, Pascal Syren, Ann-Kathrin Rahm, Florian André, Norbert Frey, Patrick Lugenbiel, Dierk Thomas

**Affiliations:** 1 Department of Cardiology, Medical University Hospital Heidelberg, Im Neuenheimer Feld 410, 69120 Heidelberg, Germany; christine.mages@med.uni-heidelberg.de (C.M.); heike.gampp@gmx.de (H.G.); pascal.syren@med.uni-heidelberg.de (P.S.); ann-kathrin.rahm@med.uni-heidelberg.de (A.-K.R.); florian.andre@med.uni-heidelberg.de (F.A.); norbert.frey@med.uni-heidelberg.de (N.F.); patrick.lugenbiel@med.uni-heidelberg.de (P.L.); 2 Heidelberg Center for Heart Rhythm Disorders (HCR), University Hospital Heidelberg, Im Neuenheimer Feld 410, 69120 Heidelberg, Germany; 3 German Centre for Cardiovascular Research (DZHK), Partner Site Heidelberg/Mannheim, University of Heidelberg, Im Neuenheimer Feld 410, 69120 Heidelberg, Germany

**Keywords:** dilated cardiomyopathy, ion channel, remodeling, sudden cardiac death, ventricular arrhythmia

## Abstract

Ventricular arrhythmias contribute significantly to morbidity and mortality in patients with heart failure (HF). Pathomechanisms underlying arrhythmogenicity in patients with structural heart disease and impaired cardiac function include myocardial fibrosis and the remodeling of ion channels, affecting electrophysiologic properties of ventricular cardiomyocytes. The dysregulation of ion channel expression has been associated with cardiomyopathy and with the development of arrhythmias. However, the underlying molecular signaling pathways are increasingly recognized. This review summarizes clinical and cellular electrophysiologic characteristics observed in dilated cardiomyopathy (DCM) with ionic and structural alterations at the ventricular level. Furthermore, potential translational strategies and therapeutic options are highlighted.

## 1. Introduction: Characteristics of Ventricular Arrhythmias in Patients with Dilated Cardiomyopathy

Ventricular arrhythmias contribute significantly to morbidity and mortality in patients with cardiomyopathies. Dilated cardiomyopathy (DCM) is one major cause of progressive heart failure (HF). The disease entity summarizes a variety of heterogenous clinical subgroups [1,2,3]. DCM is currently defined as left ventricular or biventricular dilatation and systolic dysfunction in the absence of abnormal loading conditions or coronary artery disease sufficient to cause global cardiac impairment [4,5]. The reported prevalence is estimated between 1:250 and 1:500 [1]. One-year mortality ranges between 25 and 30%, and five-year mortality yields up to 50% [2]. Cases of sudden cardiac death (SCD) due to life-threatening ventricular arrhythmias such as ventricular tachycardia (VT) and ventricular fibrillation (VF), as well as bradyarrhythmia, are reported in up to 12% of patients and account for approximately 30% of overall mortality [2,3,6].

DCM is classified either according to the European Society of Cardiology (ESC) as genetic or non-genetic or according to the American Heart Association (AHA) as secondary or primary with genetic, acquired or mixed cause, respectively [5,7]. Primary forms of DCM primarily affect the cardiac muscle, while secondary forms are caused by systemic conditions, with a large overlap between these forms [2]. Both variants may include genetic mutations, infections, autoimmune diseases, exposure to toxins and endocrine or neuromuscular causes [3]. Channelopathies (short and long QT syndrome, Brugada syndrome and catecholaminergic polymorphic ventricular tachycardia) may also be considered cardiomyopathies because of electric myocyte dysfunction [1]. Both genetic predisposition and environmental factors play a pivotal role in the natural history of the diseases.

Pathophysiological changes in DCM include a reduction in stroke volume and in cardiac output, as well as an increase in end-diastolic pressure. Compensatory volume overload results in an increased preload, contributing to increased afterload and ultimately left ventricular elevated wall stress. Furthermore, neurohumoral activation enhances sympathetic adrenergic activity and the activation of the renin–angiotensin–aldosterone system (RAAS) [3]. Clinical manifestations of patients with DCM are heterogenous and depend on etiology, age, comorbidities and the severity of the disease. Typical clinical findings include symptoms of acute or chronic HF that range from signs of volume overload, dyspnea and fatigue, to arrhythmia manifesting as palpitations, tachycardia or cardiogenic shock.

**Figure 1:**
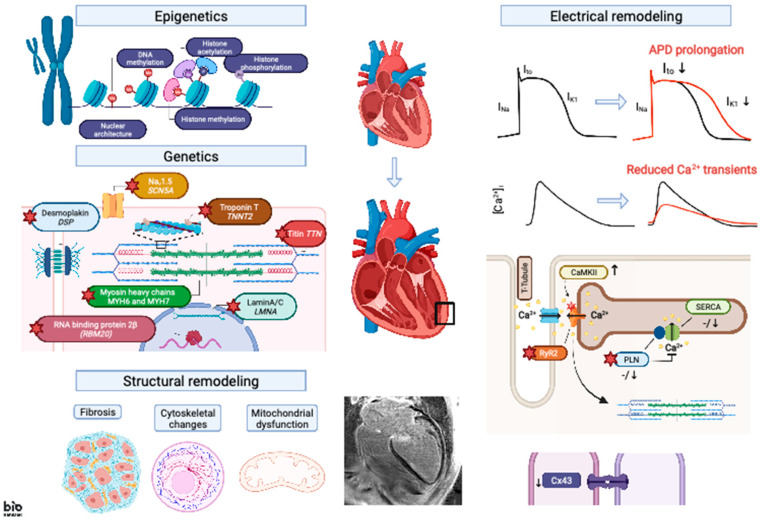
Graphical abstract on electrical ventricular remodeling in dilated cardiomyopathy.

Electrocardiographic findings are mostly unspecific and include T-wave inversion, right and left bundle branch block or atrioventricular and intraventricular conduction abnormalities [3,8]. In addition, supraventricular and ventricular arrhythmias are clinically relevant in patients with DCM [9,10,11,12,13]. This work will focus on ventricular arrhythmias (see Figure 1). The pathophysiology of arrhythmogenesis in DCM is incompletely understood despite its clinical and prognostic significance. Potential proarrhythmic mechanisms include changes in the conduction system through dilatation and the increased wall stress, the generation of arrhythmogenic substrates through focal fibrosis and neurohumoral activation leading to electrophysiological and structural remodeling [14]. Virtually all (>90%) DCM patients exhibit premature ventricular contractions (PVC), and non-sustained VT is found in 40–60% of patients’ Holter recordings [15]. These clinical findings highlight the need to better understand genetic, epigenetic and structural changes underlying ventricular arrhythmogenesis. Mechanistic insights may serve as a basis for the development of therapies to prevent maladaptive electrical remodeling and to identify patients at risk of SCD. A systematic search through the web-based engine PubMed was conducted in order to identify all studies meeting the eligibility criteria using the search terms “dilated cardiomypathy, cardiomyopathy, DCM, arrhythmia”.

## 2. Genetic Basis of Ventricular Arrhythmogenesis in DCM

More than 60 genes encoding for sarcomere proteins, cytoskeleton, nuclear envelope, sarcolemma, ion channels and/or intercellular junction molecules have been implicated in the pathogenesis of DCM to date [16,17]. The identification of new genes or novel mutations and variants in known genes is currently a subject of intensive research. Familiar forms of DCM with associated mutations are found in 30–40% of all patients, with titin (*TTN*) being the most prevalent (20–25% of familial DCM cases), followed by lamin A/C (*LMNA*, 5–10%) [17,18,19]. Genetic forms of DCM, which are typically characterized by cardiac conduction disorders and are particularly prone to malignant arrhythmias, are linked to mutations in lamin A/C (*LMNA*), cardiac sodium channel Na_v_1.5. (*SCN5A*)*,* filamin C (*FLNC*), desmoplakin (*DSP*), phospholamban (*PLN*) and RNA-binding motif protein 20 (*RBM20*) (see Table 1) [17,20]. The recognition of DCM with electric instability is clinically relevant as the presence of mutations may impact therapeutic management [17,21]. However, in many cases, a correlation between genotype and phenotype cannot be readily established due to incomplete penetrance and variable expression of the disease [16]. Furthermore, there is an overlap between ‘arrhythmogenic DCM’ and the current concept of arrhythmogenic cardiomyopathies that requires further clarification [10,22]. Direct effects of DCM-related mutations on action potential duration (APD) are not well characterized. Indeed, DCM is not characterized by a single pattern of electrophysiological changes. Although arrhythmias are commonly caused by variations in ion channels when isolated, they may also be a part of a complex manifestation of cardiomyopathy. On a cellular level, proarrhythmic defects in cardiac electrophysiology may be caused by ion channel remodeling, intercellular uncoupling, altered calcium homeostasis and changes in the extracellular matrix, each resulting or participating in dysregulated action potential duration and/or propagation [23]. For example, the defective force hypothesis proposes that genetic mutations in DCM may impair the highly organized cytoskeleton and sarcomere architecture of the cardiomyocyte, resulting in myocyte dysfunction and in arrhythmias based on structural remodeling [3,19]. Furthermore, electrophysiological defects associated with *SCN5A* gain-of-function may induce triggered activity during repolarization or diastole, whereas *SCN5A* channel loss-of-function may promote arrhythmogenesis through conduction slowing and re-entry. By contrast, pathways that cause ventricular dilatation and dysfunction associated with *SCN5A* mutations and their underlying structural defects are unclear [24].

## 3. Electrical Remodeling

DCM is characterized by complex changes in electrical properties of ventricular cardiomyocytes that predispose to ventricular arrhythmias. Electrophysiological changes include the prolongation of APD by changes in repolarization, a decrease in conduction velocity and disturbed excitation–contraction (EC) coupling. At the cellular and molecular levels, alterations involve ion channels, calcium handling proteins and intercellular gap junctions. Conduction slowing may arise from reduced depolarizing current and reduced intercellular coupling by gap junctions.

### 3.1. APD Changes

Prolongation of the action potential is a characteristic feature of cells isolated from failing animal and human hearts, irrespective of their etiology, and has been confirmed repeatedly for DCM [25,26,27]. A prolonged action potential is associated with a significant delay in repolarization, which may increase susceptibility to malignant arrhythmias by mechanisms such as triggered activity or reentry. The fast depolarization of cardiomyocytes (phase 0 of the ventricular action potential) is initiated by the opening of voltage-dependent sodium channels, which are primarily composed of Na_v_1.5. In animal models of DCM, sodium current densities did not differ from the control [28,29]. As alterations in the ion channel gene *SCN5A* are associated with DCM, there is evidence of sodium channel involvement in dilation etiology, but the mechanisms by which the disruption of sodium channel function leads to dilation remodeling remain unclear (Olson et al. 2005) [30]. *SCN5A* deficiency in a mouse model reduced membrane excitability and the resulting slowed conduction may promote arrhythmia as a result of functional block [31]. Sodium channels inactivate during repolarizing phase 1, and the transient outward potassium current (I_to1_) formed by K_v_4.2, K_v_4.3 and K_v_1.4 is activated. In a mouse model of familial DCM, I_to_ was significantly reduced in DCM cardiomyocytes before the onset of HF, and the downregulation of K_v_4.2 was evident on the mRNA and protein level [32]. In humans, two isoforms of K_v_4.3, K_v_4.3-S and K_v_4.3-L have been described, and isoform-specific remodeling was detected in failing hearts due to DCM, with increased K_v_4.3-L and reduced K_v_4.3-S mRNA transcript levels [33]. As this finding was also confirmed for ICM, it may be a common feature of remodeling in cardiomyopathies [34]. Ventricular cardiomyocytes exhibit an inward rectifying potassium current (I_K1_) that contributes to phase 3 repolarization of ventricular action potentials and to the maintenance of the negative resting membrane potential. It has been shown that the late repolarization phase for DCM is slower than that for ICM, resulting in action potential prolongation [26]. In failing hearts due to underlying DCM, the expression of the inwardly rectifying potassium channels (K_ir_) K_ir_2.2 and K_ir_2.3 decreased, which may account for the decreased I_K1_ current in DCM) [35].

### 3.2. EC Coupling

In DCM, excitation–contraction coupling is disturbed by alterations of the myocardial architecture and by expression changes of calcium-handling proteins with subsequent abnormal calcium cycling. These alterations contribute to reduced calcium transients, impaired contractility and arrhythmia. Dyads formed by the apposition of transverse (T)-tubules and junctional sarcoplasmic reticulum (jSR) are the main site for the coupling of excitation and contraction. T-tubules are invaginations of the cardiac sarcolemma with a high density of voltage-gated L-type Ca^2+^ channels (LTCC). Membrane depolarization leads to Ca^2+^ influx into the dyadic cleft via LTCC, triggering Ca^2+^ release from the SR via ryanodine receptors (RyR2), which then initiates sarcomere contraction. T-tubule remodeling is seen in many forms of HF, including DCM [36]. In DCM, there is a regional variability in the extent of T-tubule remodeling, as regions with near-normal contractility featured intact T-tubules, while regions with diminished contractility showed loss and disorganization of the T-tubule system. Furthermore, spatial re-organization occurs, with a change in T-tubule orientation from a transverse to an axial direction [37,38]. Ryanodine receptors are regulated by calcium and calcium/calmodulin dependent kinase II (CaMKII), and increased phosphorylation has been shown in patients with DCM but not in patients with ICM [39]. Increased CaMKII phosphorylation of RyR2 is suggested to play a critical role in the development of pathological diastolic SR Ca^2+^ release events (SR Ca^2+^ leaks) and the manifestation of arrhythmias [40,41,42].

Calcium sequestration into the sarcoplasmic reticulum (SR) lumen by the SR Ca^2+^-ATPase (SERCA) determines the rate of cardiac relaxation and the calcium load available for following contraction. SERCA pump activity is reversibly inhibited by PLN. Studies have described decreased expression of PLN and SERCA2 mRNA levels in human failing hearts due to DCM, while other studies show controversy about a corresponding decrease in protein levels [43,44,45,46]. Despite no significant protein level change in some studies, Ca^2+^ uptake activity and SERCA activity were shown to be significantly decreased, indicating that additional regulatory factors that have not been discovered yet may be responsible for the impaired uptake of Ca^2+^ into the SR [44]. Furthermore, genetic variants for *PLN* have been identified that may predispose to an arrhythmogenic phenotype [47,48], reflected by higher incidences of ICD therapy, premature ventricular contractions during Holter monitoring and positive family history for SCD compared with DCM patients without a *PLN* mutation [49,50]. Dominant-negative effects of *PLN* mutations on SERCA activity have been reported, resulting in decreased calcium storage and Ca^2+^ transients [51,52].

### 3.3. Cell–Cell Coupling

Gap junctions connect two neighboring cardiomyocytes at their intercalated discs and play a crucial role in impulse propagation across the myocardium and electrical synchronization between myocytes. Connexin 43 (Cx43) is the major connexin protein found in ventricular gap junctions and is predominantly expressed in its phosphorylated form in the healthy heart. Left ventricular tissue samples from patients with DCM showed a decrease in Cx43 expression and phosphorylation. Gap junctions were heterogeneously redistributed to the lateral cell borders of cardiomyocytes [53,54]. Gap junction remodeling appears to be associated with the presence of ventricular arrhythmia in DCM patients, as the patient group with a history of VT showed reduced and more heterogeneous distribution of Cx43 than the non-VT group [53]. In knockout mice, Cx43 deficiency correlates with slowed and dispersed impulse conduction [55,56], with significant contributions from changes in the phosphorylation status and subcellular distribution [27]. It has been suggested that the concurrent development of fibrosis is a prerequisite for conduction slowing [57]. Regional uncoupling due to gap junction remodeling is hypothesized to drive the dispersion of repolarization between transmural layers and contribute to electrophysiological heterogeneity of action potential duration [58]. Mechanisms by which dilation-induced cellular changes form a substrate for ventricular arrhythmias remain poorly understood. As DCM may arise from a variety of underlying causes and frequently presents with HF, distinguishing specific electrophysiological remodeling that can be attributed to DCM from alterations generally found in HF diseases is challenging.

## 4. Modulation of Epigenetic Signaling in DCM

Chromosomal alterations without changes in DNA sequence, also referred to as epigenetic changes [59,60], affect electrical remodeling in DCM [61]. Chromatin compaction via histone modifications represents one epigenetic mechanism. Typical modifications include acetylation, methylation and phosphorylation. An equilibrium of enzymes regulates the frequency of these changes. In human DCM, multiple alterations in the expression of histone methylation- [62,63,64,65], acetylation- [63,66,67] and phosphorylation- [68,69] modifying enzymes have been reported. Effects on several major pathways involved in DCM-induced electrical remodeling have been described. Histone demethylases JMJD1A, JMJD2A and JMJD2B mediate DCM-induced reactivation of atrial and brain natriuretic peptide (ANP and BNP), with reduced histone methylation in their respective promotor regions [63]. This demethylation is furthermore conveyed by increased nuclear export and reduced total expression of histone deacetylase 4 (HDAC4) [63]. While the exact effects of ANP and BNP on human cardiac electrophysiology are not fully understood due to their pleiotropic mechanism of action and high inter-species variability, relevance in this field is likely [70,71,72]. Some evidence points towards an AP-shortening in human ventricles, potentially due to influence on calcium and potassium channels [70,71,73,74]. In a porcine HF model, HDAC2 was downregulated in HF with increased ventricular effective refractory periods and prolonged QT intervals, potentially linked to reduced potassium channel transcripts *KCNJ2*, *KCNJ5* and *KCNH2* [67].

Nuclear CaMKII with relevance in electromechanical coupling and calcium handling [75] regulates chromatin accessibility via histone phosphorylation [68,69] and influences the nuclear export of HDAC4, -5 and -9 [76,77,78,79]. Bromodomain-containing protein 4 (BRD4), important for the recognition of histone acetylation sites, leads to the formation of super-enhancers with enrichment at calcium-handling gene loci [80]. Histone methylation-induced expressional changes in structural proteins such as cell adhesion molecules [81] and dystrophin [62] were reported in human DCM and show effects on AP duration [82]. Further epigenetic alterations in DCM include direct DNA methylation [83,84,85,86], changes in the higher order of chromatin [87] and interactions with the nuclear membrane. Lamin A/C mutations cause diminished conduction velocity, reduce action potential duration and *I*_Na_ current and downregulate Na_V_1.5 channel expression by the binding of lamin to the promotor region of *SCN5A* [88]. Furthermore, some definitions of epigenetics include non-coding RNAs [89] as well, with several reported alterations in DCM [61].

Several clinically used HDAC inhibitors exert QT interval-prolonging effects [90,91,92,93]. QT prolongation is likely caused by (epi-)genetic regulation and not induced by direct pharmacological interaction [94], which underlines the electrophysiological relevance of epigenetic modulation in the electrical remodeling in DCM and its therapeutic potential.

## 5. Structural Remodeling

Structural remodeling in DCM is accompanied to a variable extent by ventricular fibrosis and scar formation that can promote arrhythmia by re-entry mechanisms. Myocardial fibrosis on a cellular level is caused by increased myofibroblast activity and the deposition of extracellular matrix proteins. Various cell types and proteins are involved in these processes, and depending on the genetic causes and pathophysiology of fibrogenesis, disease progression may vary. DCM induced by sarcomere protein mutations most commonly involves myosin and troponin, causing cardiomyocytes’ degeneration and interstitial fibrosis [95]. The most common form of familial DCM caused by truncating titin mutations disturbs mitochondrial energetic metabolism and alters the cytoskeleton, thereby leading to cardiomyocyte dysfunction and inflammation and finally to myocardial fibrosis [96]. In patients with lamin A/C mutations, increased production of fibronectin, syndecans and nidogens and TGF-ß activation has been reported, with pronounced influence on cardiac electrophysiology [97].

**Figure 2:**
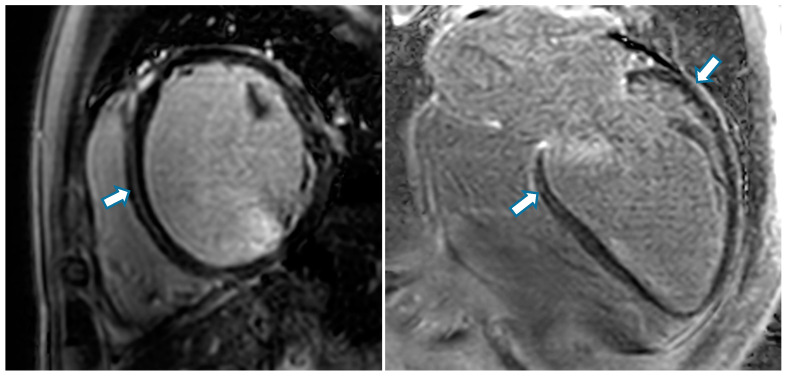
Cardiovascular magnetic resonance (late gadolinium enhancement sequences) shows subepicardial late gadolinium enhancement (white arrows) in axial (left) and longitudinal view (right).

From a clinical perspective, fibrosis has been suggested as a prognostic marker [98,99,100,101]. Currently, the main detection methods for myocardial fibrosis rely on cardiovascular magnetic resonance (CMR) with late gadolinium enhancement (LGE) and mapping techniques as the non-invasive gold standard for the identification and the quantification of myocardial fibrosis (see Figure 2). While LGE is ideal for the detection of focal fibrosis, native T1 mapping and extracellular volume (ECV) quantification using gadolinium contrast agent are more suitable for the detection of diffuse fibrosis and may therefore better detect early stages of DCM [102].

In most patients with sustained and hemodynamically not tolerated VT, MRI is limited by artifacts from implanted ICDs for secondary prevention, and although scar detection and ECV imaging by cardiac CT are possible, data are still scarce compared to CMR, and it is not performed in clinical routine [103,104]. Endomyocardial biopsy is used in selected cases in clinical routine, and serum markers are under evaluation in clinical studies [105]. The analysis of LGE patterns render the identification of patients with non-ischemic causes possible, as those patients show non-ischemic patterns including mid-wall/sub-epicardial or patchy distribution, in contrast to ischemic patterns in cardiomyopathies [106].

Fibrosis itself has been identified as a risk factor for mortality in DCM patients as the presence of LGE has been associated with increased VT occurrence and with overall mortality [107,108]. In addition, the complexity and extent of myocardial scars have been associated with VT incidence and mortality [109,110]. The computer-based modeling of reentry circuits in individual patients may help to identify patients at risk for arrhythmia and support the planning of catheter ablation strategies [111].

## 6. Translational Perspective and Conclusions

The individualized risk prediction and treatment of patients with DCM remain challenging. The optimal pharmacological treatment according to current guideline recommendations forms the basis of DCM therapy [9,112]. Yet, the current treatment of HF in DCM does not differ from general HF management and effects of disease-modifying drugs on ionic and structural alterations in DCM are less well characterized.

In antiarrhythmic therapy, potential reversible causes need to be excluded first (such as the existence/progress of coronary artery disease, changes in electrolytes or proarrhythmic drugs). Secondly, the severity and treatment options of the arrhythmia itself (PVC vs. VT/VF), as well as the need for additional device therapy such as ICD therapy, need to be addressed [10]. Antiarrhythmic drugs in DCM are mostly limited to class III compounds acting through action potential prolongation. Antiarrhythmic treatment should also consider using secondary antiarrhythmic effects of not primarily antiarrhythmic drugs such as sacubitril/valsartan [113]. Furthermore, only recently a potential antiarrhythmic effect of the sodium-glucose cotransporter 2 inhibitor dapagliflozin has been reported [114]. Recently, the Defibrillator Implantation in Patients with Nonischemic Systolic Heart Failure Trial (DANISH-Trial) found no long-term benefit of prophylactic ICD implantation among a heterogenic cohort of patients with non-ischemic HF [115]. Therefore, it appears relevant to identify DCM patients with the highest risk of SCD. The ‘arrhythmic risk stratification in nonischemic dilated cardiomyopathy’ trial (ReCONSIDER) aims at evaluating a two-step model including non-invasive risk factors and electrophysiology data for risk stratification in DCM [116].

Some ECG features could be clues of specific DCM subtypes, as certain disease-causing genes are associated with characteristic ECG abnormalities and indeed point toward particularly aggressive forms [8]. Currently, genetic testing is not part of routine patient care in DCM because of high cost and relatively low yield, but might be considered in patients suspected to have arrhythmogenic cardiomyopathy involving *LMNA*, *PLN* or *FLNC* mutations [10]. A genetic test is generally performed in an index patient with either a clinical diagnosis that fulfills the clinical criteria for the disease in question or when there is at least a reasonable indication for the presence of that specific disorder [10]. Emerging data suggest that genetic information may allow for gene-specific, more personalized therapeutic strategies. In an individualized medical approach, it is therefore relevant to evaluate every patient individually.

The focus in risk stratification is shifting towards optimized characterization of the underlying etiology of DCM and the development of multi-parametric models [117]. Advanced SCD risk stratification could include the underlying DCM pathology or family history for ventricular arrhythmias, clinical presentation, results of cardiac MRI, echocardiography and ECG. Recently, sex- and age-based differences in the natural history of DCM have been reported [118]. Lower baseline left ventricular ejection fraction (LVEF), higher New York Heart Association (NYHA) class (III–VI), significant mitral regurgitation, the presence of left bundle branch block and higher natriuretic peptide levels are predictors of adverse outcomes [119].

In the case of refractory VT, catheter ablation should be considered depending on the suspected arrhythmogenic substrate and scar evaluation to reduce VT/VF burden [9]. Integrating imaging-based ablation strategies and computational modeling may improve ablation strategies, ICD risk prediction and outcome [111,120]. In the future, the multimodal characterization of patients is expected to provide the basis for optimized, personalized antiarrhythmic DCM management.

## Figures and Tables

**Table 1:** Monogenetic causes of DCM particularly prone to ventricular arrhythmia.

Protein	Gene	Protein Function
Titin	*TTN*	Sarcomere structural protein
Lamin A/C	*LMNA*	Inner nuclear membrane
Cardiac sodium channel Na_v_1.5	*SCN5A*	Cardiac sodium channel α-subunit
Filamin C	*FLNC*	Actin cytoskeleton
Desmoplakin	*DSP*	Desmosomal protein
RNA-binding motif protein 20	*RBM20*	RNA binding and splicing regulation
Phospholamban	*PLN*	Sarcoplasmic reticulum protein involved in calcium homeostasis

## Data Availability

Not applicable.
